# Zebrafish as a platform to evaluate the potential of lipidic nanoemulsions for gene therapy in cancer

**DOI:** 10.3389/fphar.2022.1007018

**Published:** 2022-10-31

**Authors:** María Cascallar, Pablo Hurtado, Saínza Lores, Alba Pensado-López, Ana Quelle-Regaldie, Laura Sánchez, Roberto Piñeiro, María de la Fuente

**Affiliations:** ^1^ Nano-Oncology and Translational Therapeutics Group, Health Research Institute of Santiago de Compostela (IDIS), SERGAS, Santiago de Compostela, Spain; ^2^ Centro de Investigación Biomédica en Red Cáncer (CIBERONC), Madrid, Spain; ^3^ Universidade de Santiago de Compostela (USC), Santiago de Compostela, Spain; ^4^ Roche-Chus Joint Unit, Translational Medical Oncology Group, Oncomet, Health Research Institute of Santiago de Compostela, Santiago de Compostela, Spain; ^5^ Department of Zoology, Genetics and Physical Anthropology, Universidade de Santiago de Compostela, Campus de Lugo, Lugo, Spain; ^6^ Center for Research in Molecular Medicine and Chronic Diseases (CIMUS), Campus Vida, Universidade de Santiago de Compostela, Santiago de Compostela, Spain; ^7^ Preclinical Animal Models Group, Health Research Institute of Santiago de Compostela (IDIS), Santiago de Compostela, Spain; ^8^ DIVERSA Technologies S.L, Santiago de Compostela, Spain

**Keywords:** zebrafish, nanomedicine, gene therapy, miRNA, plasmid, cancer

## Abstract

Gene therapy is a promising therapeutic approach that has experienced significant groth in recent decades, with gene nanomedicines reaching the clinics. However, it is still necessary to continue developing novel vectors able to carry, protect, and release the nucleic acids into the target cells, to respond to the widespread demand for new gene therapies to address current unmet clinical needs. We propose here the use of zebrafish embryos as an *in vivo* platform to evaluate the potential of newly developed nanosystems for gene therapy applications in cancer treatment. Zebrafish embryos have several advantages such as low maintenance costs, transparency, robustness, and a high homology with the human genome. In this work, a new type of putrescine-sphingomyelin nanosystems (PSN), specifically designed for cancer gene therapy applications, was successfully characterized and demonstrated its potential for delivery of plasmid DNA (pDNA) and miRNA (miR). On one hand, we were able to validate a regulatory effect of the PSN/miR on gene expression after injection in embryos of 0 hpf. Additionally, experiments proved the potential of the model to study the transport of the associated nucleic acids (pDNA and miR) upon incubation in zebrafish water. The biodistribution of PSN/pDNA and PSN/miR *in vivo* was also assessed after microinjection into the zebrafish vasculature, demonstrating that the nucleic acids remained associated with the PSN in an *in vivo* environment, and could successfully reach disseminated cancer cells in zebrafish xenografts. Altogether, these results demonstrate the potential of zebrafish as an *in vivo* model to evaluate nanotechnology-based gene therapies for cancer treatment, as well as the capacity of the developed versatile PSN formulation for gene therapy applications.

## 1 Introduction

Over the past decades, gene therapy has flourished. The basis of this therapy is focused on the use of exogenous therapeutic nucleic acids (NAs) that have the capacity to modify the expression of disease-related genes. NAs involved in gene therapy are micro RNAs (miRs), small interfering RNAs (siRNAs), short hairpin RNAs (shRNAs), antisense oligonucleotides (ASOs) and DNA plasmids (pDNAs) (Sayed et al., 2022).

One of the main limitations in the development of novel gene therapies is the need for efficient carriers capable of protecting and transporting them to their site of action. Viral vectors are the carriers that have moved most quickly to clinical trials, due to the ability of the virus to carry and protect the genetic material to specific cells (Santiago-Ortiz and Schaffer, 2016; Mohammadinejad et al., 2020). Despite this, viral vectors accumulate several disadvantages, such as limitation in the length of the cargo (e.g., 10 kb in lentiviral vectors), insertional mutagenesis, and immunogenicity due to the antibodies against these common viruses produced throughout life (Walther aand Drugs, 2000; Amer, 2014; Mohammadinejad et al., 2020; Zu and Gao, 2021). In this sense, non-viral vectors have been proven to successfully resolve the limitations of viral vectors (Mohammadinejad et al., 2020). A clear example is nanomedicine, which arises from the application of nanotechnology in the field of biomedicine, providing several advantages for the intracellular delivery of macromolecules, such as NAs. As proof of this, in recent years several breakthroughs have taken place. In 2018, Onpattro^®^ became the first FDA-approved lipid nanoparticle for gene therapy (Hoy, 2018; Akinc et al., 2019). Additionally, in 2021, mRNA-based vaccines against Covid-19 reached the market (Corbett et al., 2020; Polack et al., 2020), opening a new era for the engineering and application of gene therapy.

Despite the successful advances of the past few years, translating more gene nanomedicines from bench to bedside is still a challenge. In this sense, the use of robust preclinical models that can better predict the future behavior of nanosystems is essential for their development and validation, improving the translation process (Wick et al., 2015; Sahlgren et al., 2017; Sieber et al., 2019b; Bouzo et al., 2020; Boix-Montesinos et al., 2021). *In vivo* models are needed to evaluate biodistribution, toxicity and efficacy, among other parameters. Rodents, the most common animal model, have multiple advantages, such as anatomical and genomic similarities to humans. Nevertheless, they entail certain disadvantages, including the high cost of maintenance and small progeny that prevents the possibility of carrying out large studies (Lieschke and Currie, 2007a; Sieber et al., 2019). A valuable alternative as an *in vivo* platform to evaluate the potential of nanomedicine is the zebrafish (Gutiérrez-Lovera et al., 2017; Pearce et al., 2018; Sieber et al., 2019; Cascallar et al., 2022).

The use of the zebrafish (*Danio rerio*) in developmental biology and genetics studies dates back to the 1970s (Streisinger et al., 1981). Since then, applications have expanded to study multiple human pathologies, such as cancer, as well as biodistribution, toxicity and pharmacological screening of new drugs (Jia et al., 2019). The success of zebrafish in research is based on their biological characteristics (Zon, 1999). Specifically, their small size enables easy handling, and large number of individuals can be maintained in optimal experimental conditions. Due to their short life cycle, the main organs develop practically within 48 h, sexual maturity is reached at approximately 3 months of life, and large offspring allow large-scale studies to be carried out (Kimmel et al., 1995). Additionally, the zebrafish reference genome has revealed that approximately 80% of the genes have a human orthologue related to diseases (Howe et al., 2013).

Based on these features, in the field of nanomedicine this model is being proposed to assess the biocompatibility and toxicity of several nanomaterials, but also to validate their therapeutic efficacy (Gutiérrez-Lovera et al., 2017; Sieber, Grossen, Bussmann, et al., 2019; Wang et al., 2019; Pensado-López et al., 2021; Cascallar et al., 2022). The presence in zebrafish of organs and metabolic pathways analogous to those of humans allows toxicological and biocompatibility evaluations, and the large number of offspring enables high-throughput and multi- and transgenerational screens (Horzmann and Freeman, 2018). In addition, the response of zebrafish to several substances has been reported to be concordant with that observed in mammalian models (Sipes et al., 2011). In the context of cancer, the transparency of embryos and the availability of fluorescently labelled transgenic zebrafish lines offer the possibility to track cancer cells in xenograft assays or genetic models and thus understand their behavior, dissemination, metastasis, extravasation, or interaction with the tumor microenvironment or immune cells (Lawson & Weinstein, 2002; Renshaw et al., 2006; Ellett et al., 2011). On the other hand, the transparency of zebrafish allows the determination of the toxicity of nanosystems in different anatomical sites of the fish and their tracking to establish biodistribution and interaction profiles with tumor cells without the need for invasive techniques (Lee et al., 2017). In this sense, transgenic zebrafish models allow for real-time tracking of tumor cells without the need to immunostain cells, thus avoiding non-specific labeling and imaging issues derived. As a consequence of the abovementioned, several nanomedicines have been developed and tested in zebrafish, including gene therapies (Wang et al., 2019; Al-Thani et al., 2021; Saraiva et al., 2021).

In our group, we have previously developed different types of nanosystems for miR-based gene therapy for cancer treatment. These nanocarriers, protamine nanocapsules and sphingomyelin-based nanosystems, demonstrated their *in vitro* potential to interfere in the cancer process (Reimondez-Troitiño et al., 2019; Nagachinta et al., 2020). On subsequent studies by our group, Lores et al. (2022) developed putrescine-sphingomyelin nanosystems (PSN) for cancer gene therapy applications establishing for the first time the use of the natural polyamine putrescine for the development of non-viral vectors, taking advantage of the cationic nature of this compound and the greater affinity of cancer cells for this type of molecules (Thomas et al., 2016; Tracy et al., 2016). In this work, a therapeutic plasmid DNA (pDNA) encoding for the Fas Ligand protein, which promotes the activation of apoptotic pathways, was associated with PSN, and the potential of the developed formulation confirmed *in vitro*, in a triple negative breast cancer cell line (MDA-MB-231), and *in vivo*, in both a zebrafish embryo xenograft model and in an orthotopic mouse model, evidencing a high correlation in terms of efficacy. Based on this data, the present work aimed to further demonstrate the potential of zebrafish embryos as an intermediate model between *in vitro* and *in vivo* mammalian models for the evaluation of novel gene therapies, using for this purpose PSN associated with two different types of nucleic acids, miR and pDNA (Lores et al., 2022).

## 2 Materials and methods

### 2.1 Materials

All the miRs used in this work (Table 1) were purchased from Eurofins Genomics (Ebersberg, Germany). Penicillin-Streptomycin, Hoechst 33342, DiI (1,1′-Dioctadecyl-3,3,3′,3′-Tetramethylindocarbocyanine Perchlorate), agarose and SYBR Gold were provided by Thermo Fisher (Massachusetts, United States). C11 TopFluor Sphingomyelin (N-[11-(dipyrrometheneboron difluoride)undecanoyl]-D-erythro-sphingosylphosphorylcholine was purchased from Avanti Polar Lipids (Alabama, United States). Brilliant III Ultra-Fast SYBR Green QPCR Master Mix Kit was acquired from Agilent Technologies (California, United States). Nuclease-free water was provided by Corning (New York, United States). NYzol reagent was purchased from NZYtech (Lisboa, Portugal). Dulbecco′s Modified Eagle′s Medium (DMEM), Phosphate Buffered Saline (PBS), Tricaine methanesulfonate, Vitamin E (DL-α-Tocopherol), N-Phenylthiourea (PTU), Polyvinylpyrrolidone (PVP), Trypsin-EDTA Solution and MOWIOL^®^ 4-88 Reagent were kindly provided by Merck (Darmstadt, Germany). Ethanol of analytical grade was purchase from VWR (Barcelona, Spain). Paraformaldehyde was provided by IESMAT (Madrid, España). Sphingomyelin (Lipoid E SM) was acquired from Lipoid GmbH (Ludwigshafen, Germany). Oleamide-modified putrescine ((9Z)-N-(4-Aminobutyl)-9-octadecenamide, CAS RN: 1005454-33-0) was provided by GalChimia (A Coruña, Spain). The plasmid pcDNA4TO-mito-mCherry-10xGCN4_v4 was purchased in AddGene (Plasmid #60914; http://n2t.net/addgene:60914; RRID:Addgene_60914) (Massachusetts, United States).

**TABLE 1:** compilation of sequences of the miR used in this work.

	Sequence
miR control	5′CAG​UAC​UUU​UGU​GUA​GUA​CAA3′
miR control-Cy5	5′Cy5-CAGUACUUUUGUGUAGUACAA3′
miR 145	5′GUC​CAG​UUU​UCC​CAG​GAA​UCC​CU3′

### 2.2 Formulation of the nanosystems and nucleic acid association

As previously described (Lores et al., 2022), putrescine nanosystems were formulated by ethanol injection method. Briefly, 5 mg of vitamin E (VitE), 0.5 mg of sphingomyelin (SM) and 0.25 mg of putrescine modified with oleamide (Pt) were dissolved in 100 µl of ethanol and injected under magnetic stirring at 700 RPM in 1 ml of Molecular Grade Water. The suspension was kept under stirring at room temperature for 5 min. Then, 5 µg of miR were dissolved in 100 μl of H2O nuclease-free and added over 100 µl of preformed nanocarriers, for 20 min under magnetic stirring at 500 RPM to achieve the association.

Moreover, previously to the pDNA-Cy5 association with the PSN, it was labelled with Cy5 with the Label IT^®^ TrackerTM Intracellular Nucleic Acid Localization Kit (Mirus Bio, Madison, United States).

### 2.3 Physicochemical characterization

Physicochemical characterization of the nanosystems were performed using a Zetasizer^®^ Nano ZS (Malvern Instruments, England), which provides mean size, polydispersity index (PdI) and zeta potential (ZP). Dynamic light scattering (DLS) allows to perform size and PdI measurements of samples previously diluted 1:10 in MilliQ water. Samples were analysed in disposable microcuvettes (ZEN0040, Malvern Instruments) with a detection angle of 173° at room temperature. Laser Doppler anemometry (LDA) allows to evaluate ZP using folded capillary cells cuvettes (DTS 1070, Malvern Instruments) and a 1:40 diluted sample in MilliQ water.

### 2.4 Association efficiency

An 3% agarose gel electrophoresis was performed to evaluate the association efficiency of the miR. A known amount of miR (2 µg) was mixed with Loading buffer, Tris-Borate-EDTA (TBE) buffer and SYBR Gold. The agarose gel was prepared in TAE buffer, composed by Tris, acetic acid and EDTA 0.5 M. Prepared samples were loaded, and the gel was run at 80 V for 40 min, making use of a Mini-Sub Cell GT Cell (BioRad, California, United States). The result was evaluated with the ChemiDocTM MP Imaging System (Bio-Rad, California, United States), in which not-associated miR appears as a band in the gel. In the case of pDNA, 0.2 µg was loaded in a 1% agarose gel, following the same protocol.

### 2.5 miR-145 effects in sox9b and gata6 expression-zebrafish as a feasible model for gene therapy

#### 2.5.1 Zebrafish husbandry and microinjection

Zebrafish embryos were obtained by mating wild type adults, which were maintained in 30-L tanks with a 14 h/10 h light/dark cycle and a temperature of 28.5°C. Embryos of 0 h post fertilization (hpf) were collected, placed in 90 mm × 15 mm Petri dishes, and subsequently microinjected with 1–3 nl of free miR Control, free miR145, PSN alone, PSN/miR Control or PSN/miR 145 (0.25 μg/μl). Microinjected embryos as well as controls were kept at 28.5°C until 72 hpf. All the procedures described for zebrafish were performed in agreement with the Animal Care and Use Committee of the University of Santiago de Compostela and the standard protocols (Directive 2012–63-UE).

#### 2.5.2 Real-time quantitative polymerase chain reaction

Real-time quantitative polymerase chain reaction (RT-qPCR) was performed with three biological replicates (10 embryos/pool) and three technical replicates for each. Total RNA was isolated from the embryos with the NYzol reagent and the purification was based on a phenol-chloroform protocol. Reverse transcription was performed with the AffinityScript Multiple Temperature cDNA Synthesis Kit (Agilent) following the manufacturer’s protocol. RT-qPCR was performed using the Brilliant III Ultra-Fast SYBR Green QPCR Master Mix Kit and the Stratagene Mx3005P Thermal Cycler (Agilent Technologies). To analyze the expression levels, the ΔΔCT method was applied, using the actb2 gen as housekeeping and statistical analyses were performed in SPSS Statistics (IBM) through a T Student test. Statistical significance was considered if *p* < 0.05. The actb2 primers (Forward: ACT​TCA​CGC​CGA​CTC​AAA​CT; Reverse: ATC​CTG​AGT​CAA​GCG​CCA​AA) were designed using Primer BLAST (Altschul et al., 1990), while those for sox9b (Forward: AGC​TCA​GCA​AAA​CAC​TCG​GC; Reverse: CCG​TCT​GGG​CTG​GTA​TTT​GT) (Steeman et al., 2021) and gata6 (Forward: AAA​CCT​CAG​AAG​CGC​ATG​TC; Reverse: AGA​CCA​CAG​GCG​TTG​CAC) (Zeng et al., 2009) were obtained in the literature.

### 2.6 Cell culture

MDA-MB-231 (CRM-HTB-26™) triple negative breast cancer cell line and MCF7 (HTB-22™) brest cancer cell line were obtained from the American Type Cell Culture (ATCC). Cells were cultured in Dulbecco′s Modified Eagle′s Medium (DMEM) - high glucose, supplemented with 10% Fetal bovine serum and 1% penicillin/streptomycin. Cells were maintained in a humid atmosphere (95%), 5% of CO_2_ and 37°C.

### 2.7 Cellular uptake

Internalization assays were performed on MDA-MB-231 cells to evaluate nanoemulsions labelled with sphingomyelin TopFluor^®^ (4.5 µg/nanoemulsion). Cells were seeded on an 8-well chambered slide at 40.000 cell/well. After 24 h of incubation at 37°C, cells were washed with PBS and 200 µl of non-supplemented DMEM were added per well. Nanocarriers with and without associated miR-Cy5 were mixed in each well at a concentration of 0.2 mg/mll. Cells were incubated with the nanocarriers for 4 h at 37°C. After this time, cells were washed twice with PBS and fixed with 4% (w/v) paraformaldehyde for 15 min. Cells were again washed twice with PBS and cellular nuclei were stained with Hoechst (0.01 mg/ml in PBS) for 5 min in darkness at room temperature. After that, cells were washed 3 times for 5 min with PBS, which was aspirated after the last wash. Then, walls were removed and Mowiol^®^ 4-88 was added for placing a coverslip; after that, samples were kept drying in darkness overnight. Uptake results were evaluated by confocal microscopy (SP8 Laser Microscope, Leica).

### 2.8 Zebrafish maintenance

Wildtype zebrafish embryos were maintained in E3 medium with 1-phenyl 2-thiourea (PTU) at 28.5°C. E3 is a saline medium composed by NaCl, KCl, CaCl_2_ · _2_H_2_O and MgSO_4_ (Murphey and Zon, 2006), traditionally used for maintaining the embryos, whereas PTU is a compound that inhibits the melanogenesis (Karlsson et al., 2001), maintaining the transparency of embryos for a longer time and avoiding the pigmentation, which could interfere later in confocal microscopy. PTU was only used in the assays evaluated by confocal imaging to improve the transparency of the embryos.

### 2.9 *In vivo* uptake

Nanoemulsions labelled with TopFluor were incubated with the embryos in a 96-well plate with a final volume of 100 µl per well, for 72 h at 34°C. The lipidic concentration used in these assays was 0.5 mg/ml and the concentration of TopFluor was 20 μg/ml. Internalization was tested in three different conditions with at least 16 replicates per condition: Control (MilliQ water), PSN, PSN/miR, and PSN/pDNA (with Cy5-labelled miR and pDNA). Permeability was evaluated by confocal microscope after embryos suppression with tricaine overdose, fixation with paraformaldehyde 4% for 30 min, and wash with PBS twice.

Moreover, in the case of PSN and PSN/pDNA, mortality assessment was performed to evaluate the toxicity of the nanosystems. Embryos were evaluated each 24 h and mortality was observed.

### 2.10 Nanoemulsions biodistribution *in vivo*

To evaluate the biodistribution of nanoemulsions *in vivo*, 48 hpf zebrafish embryos were microinjected in the duct of Cuvier with TopFluor-labelled PSN (with and without Cy5-labelled miR/pDNA) previously concentrated 10 times by the SpeedVac Concentrator (Savant SPD111V-120, Cambridge Scientific, Massachusetts, United States). The microinjection was carried out with a binocular loupe (SMZ745, Nikon), the IM 300 Microinjector (Narishige, Tokyo, Japan), and needles made with the PC-10 Puller (Narishige, Tokyo, Japan) from glass capillaries (Harvard Apparatus, Massachusetts, United States). After 48 h from the microinjection, embryos were processed as explained in Section 2.9 and nanoemulsions biodistribution was evaluated by confocal microscopy.

### 2.11 Nanoemulsions behavior in xenografted zebrafish

In order to evaluate the behavior of the nanoemulsions in a metastatic-like *in vivo* environment, 48 hpf zebrafish embryos were xenografted with MDA-MB-231 cells, previously labelled with DiI. Cells were resuspended in PVP 2% and 200–300 cells were injected into the perivitelline space, as explained in Section 2.9. After 24 h, TopFluor-labelled PSN (with and without Cy5-labelled miR/pDNA), previously concentrated 10 times by the SpeedVac Concentrator, were microinjected into the Duct of Cuvier. *In vivo* behaviour and interaction between developed nanocarriers and cancer cells were evaluated by confocal microscopy subsequent to following the same protocol as explained in Section 2.9.

## 3 Results

### 3.1 Nanoemulsions (PSN) characterization

In this work, we wanted to evaluate the potential of zebrafish to test novel gene nanotherapies, and for that purpose, we associated two different types of NAs to versatile PSN, namely miR and pDNA. To formulate the PSN, we followed the ethanol injection method, as previously described (Bouzo et al., 2020; Lores et al., 2022). PSN have a mean size below 100 nm, a positive zeta potential (ZP) (around +60 mV) and a polydispersity index (PdI) of about 0.2 (Table 2), as determined by Dynamic Light Scattering (DLS) and Laser Doppler Anemometry (LDA).

**TABLE 2:** Physicochemical characterization of PSN with and without different miR associated by DLS and LDA**.**

Formulation	Length (bp)	Size (nm)	PdI	ZP (mV)
PSN	—	91 ± 6	0,23	+58 ± 6
PSN/miR control	21	123 ± 2	0,17	+45 ± 1
PSN/miR 145	23	108 ± 3	0,20	+40 ± 2
PSN/pDNA	6,717	164 ± 4	0,09	+44 ± 5

Abbreviations: PdI, polydispersity index; ZP, zeta potential.

**FIGURE 1:**
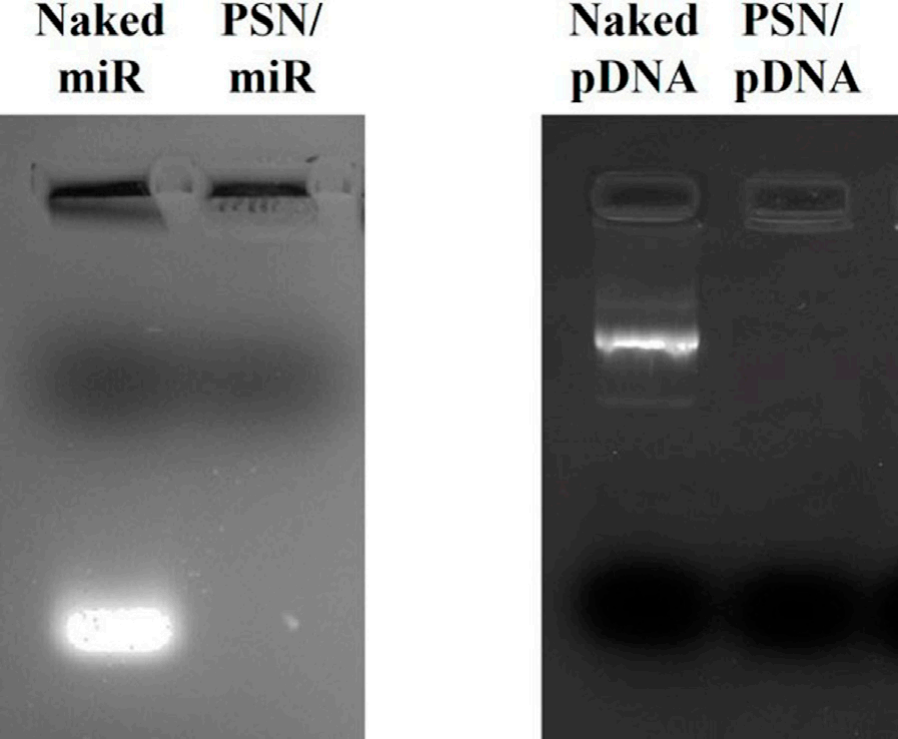
1% agarose gel electrophoresis (80 V, 40 min) to evaluate the association efficacy between PSN and nucleic acids: miR (2 µg) and pDNA (0.2 µg).

The conditions for an efficient NA association preserving the colloidal properties of the nanocarriers were conveniently adjusted. As can be observed in Table 2, in all tested conditions, an increase in the hydrodynamic size and a decrease in the ZP were observed after the association of the NA, due to the interaction between their phosphate groups and the primary amines from the putrescine. Particularly, the association of the miR showed an increase in the size of around 30 nm. After the incubation with the pDNA, a higher increase in size was observed, which could be due to the higher molecular weight of the NA (near 7,000 bp compared to the 21–23 bp of the miRs). In both cases, the resulting changes in the physicochemical parameters suggest a successful association, as was shown in other works (Liu et al., 2016; Nagachinta et al., 2020). Moreover, the PdI remained below 0.2 after NAs association, demonstrating that the PSN population is homogeneous. Even though these results indicate an efficient association of the NAs, an agarose gel electrophoresis was performed to provide additional evidence. (Figure 1). As observed, miR and pDNA were successfully retained in the well, as consequence of their interaction with PSN. Only naked NA molecules, loaded for control, freely moved in the gel.

Morover, in our previous work by Lores et al., PSN stability experiments were carried out. PSN stability under storage conditions, at 4°C, was evaluated, and the results demonstrate that they are stable for up to 21 days, according to the lack of variation in size, PdI, and ZP. Furthermore, the association between the pDNA and the PSN was studied and confirmed to remain stable by agarose gel electrophoresis. The conditions evaluated were the stability upon incubation with complete cellular medium, after incubation with DNases and after 3 months of storage at 4°C, demonstrating the high stability of the association as well as the protective role of PSN against DNases (Lores et al., 2022).

### 3.2 Transfection efficacy in the zebrafish embryo: *In vivo* effects of PSN/miR 145 in sox9b and gata6 expression

The characterization of PNS demonstrates the correct association of different NAs, however, for the PNS to exert the desired therapeutic effect, a key factor is the release of the cargo inside the cells. In this sense, zebrafish embryos allow us to evaluate *in vivo* the transfection capacity of the NAs. As mentioned before, zebrafish compile characteristics that make them highly appropriate to evaluate gene therapies. In this specific case, embryo robustness, their external fecundation, and the ease with which they are genetically manipulated make zebrafish the ideal model for this kind of assessment.

With the aim of studying the correct release of the associated NAs inside the cells, miR 145 was chosen due to its effect on gene expression in the zebrafish embryo. This miR is known to downregulate sox9b and gata6 genes when overexpressed in zebrafish (Zeng et al., 2009; Steeman et al., 2021). Embryos of 0 hpf, one-cell stage, were microinjected with PSN associated and non-associated with miR (Control and 145), and with free miR (Control and 145). The chosen stage to start the treatment was 0 hpf to potentially modulate the genes during the first cell division and avoid possible interference in successive stages. Furthermore, microinjection was the selected method to ensure the introduction of the PSN/miR 145 into zebrafish embryos.

**FIGURE 2:**
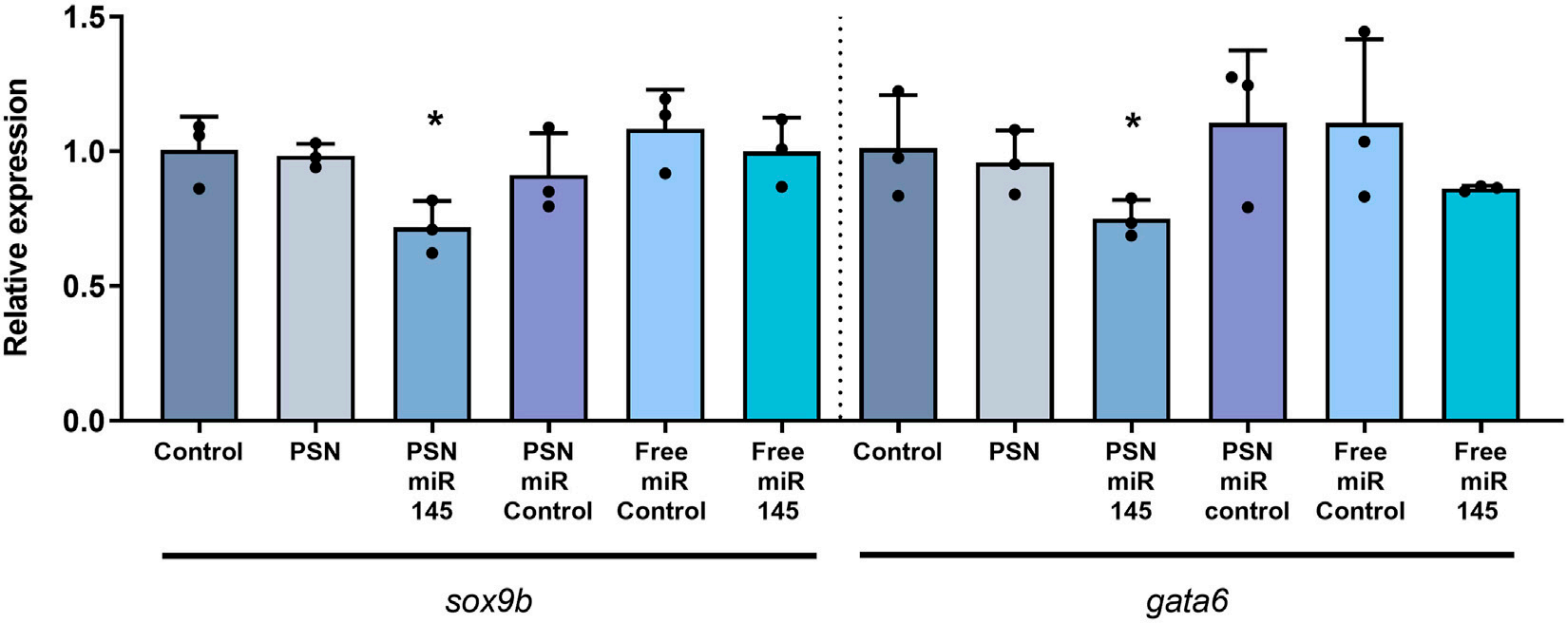
RT-qPCR results of the effect of miR 145 associated and non-associated with PSN in the relative expression of sox9b and gata6 genes in zebrafish embryos.

In order to determine if the microinjected PSN miR145 or the free miR145 were able to modify sox9b and/or gata6 expression, a RT-qPCR was performed 3 days later (72 hpf). In accordance with previous observations (Zeng et al., 2009; Steeman et al., 2021), miR145 increase led to a significant decrease in sox9b (*p* value 0,0347) and gata6 (*p* value 0,0364) expression but only when associated in PSN (Figure 2), and not when microinjected alone, in 72 hpf zebrafish embryos. Similarly, neither free miR control nor PSN alone nor PSN/miR control were able to modify their expression.

### 3.3 PSN/miR-pDNA *in vivo* uptake

Zebrafish is also characterized by being transparent in their first embryonic stages, and this fact allows us to evaluate fluorescent-labelled compounds as well as cells and nanoparticles (Gutiérrez-Lovera et al., 2017; Sieber, Grossen, Bussmann, et al., 2019). It is relevant that nanosystem internalization experiments based on incubation are easily performed in zebrafish embryos, however, this cannot be done in rodents, demonstrating the advantages of zebrafish as a model. Taking advance of this, an *in vivo* internalization assay was performed in 48 hpf embryos to study PSN behavior.

**FIGURE 3:**
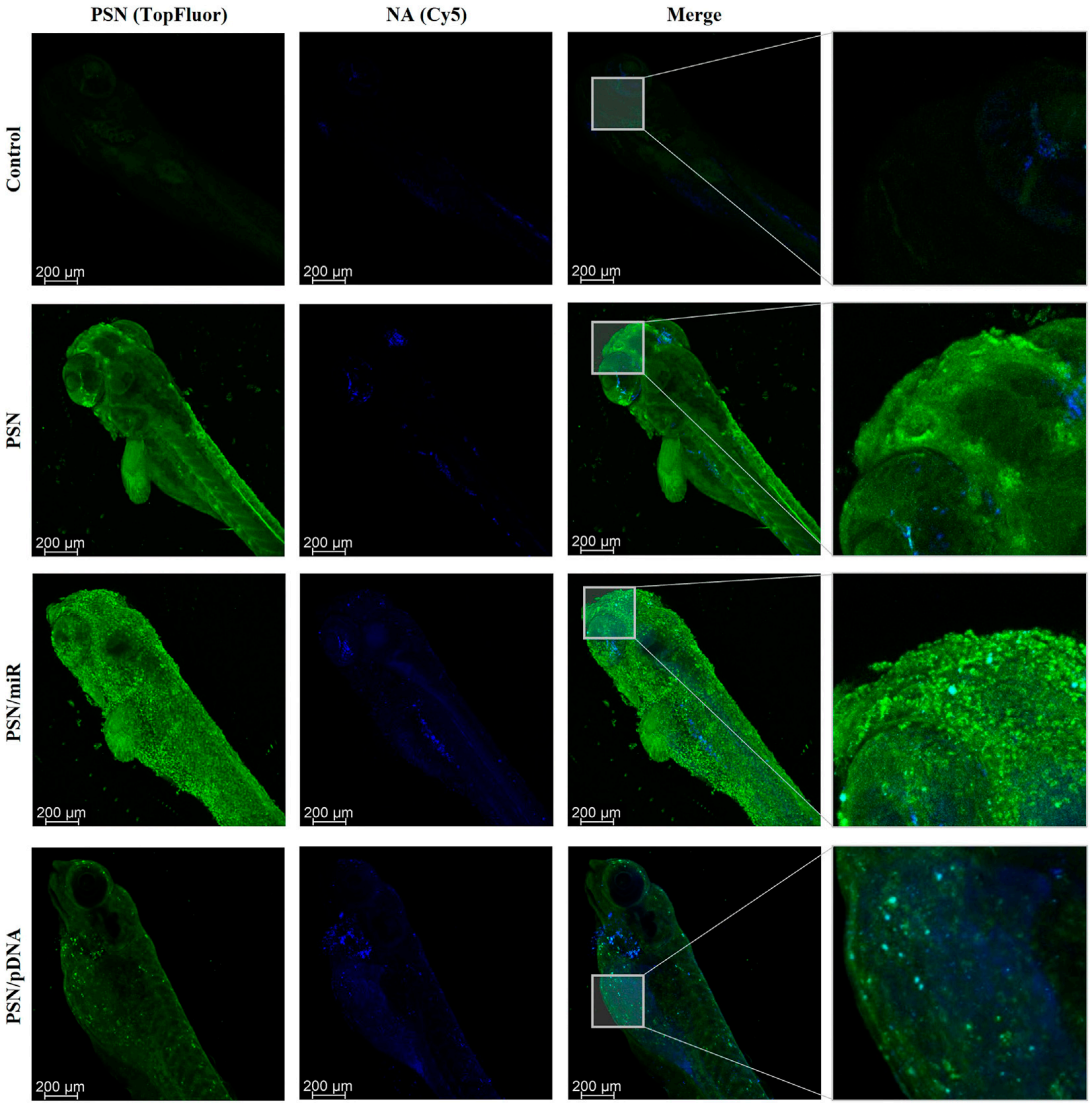
Confocal images of zebrafish embryos after a 72 h incubation with TopFluor-PSN (in green) associated and non-associated with Cy5-labelled miR and pDNA (in blue).

Cy5-labelled miR and pDNA were respectively associated with fluorescent PSN (labelled with TopFluor®-sphingomyelin). The use of zebrafish embryos for this type of assay allows us to easily incubate NA-loaded PSN in their media, in this case, 72 h. The results, which were obtained by confocal microscopy, demonstrated a high internalization by cells of the fluorescent PSN, and most importantly, allowed also to determine the presence of the associated NAs, miR and pDNA (Figure 3). Furthermore, experiments confirmed the co-localization (in cyan) of PSN (in green) and NAs (in blue) in an *in vivo* model with a superior level of complexity (Figure 3). This colocalization proves that PSN and NAs remain associated during the uptake process, allowing the efficient transport of NAs into the cells, which is a key step for successful gene therapy.

Furthermore, these uptake studies demonstrated the low toxicity of the nanocarriers (Supplementary Figure S1), producing less than 20% of mortality in the embryos.

### 3.4 PSN/miR-pDNA *in vivo* biodistribution

Following the same strategy of leveraging zebrafish embryo transparency, a PSN biodistribution assay was subsequently performed. Zebrafish transparency, which lasts until 24 hpf and can be extended with the use of PTU (Karlsson et al., 2001), allowed us to demonstrate the potential of PSN to be a carrier for gene therapy since we can monitor their stability and biodistribution in the circulatory system of the fish (Sieber, Grossen, Bussmann, et al., 2019).

**FIGURE 4:**
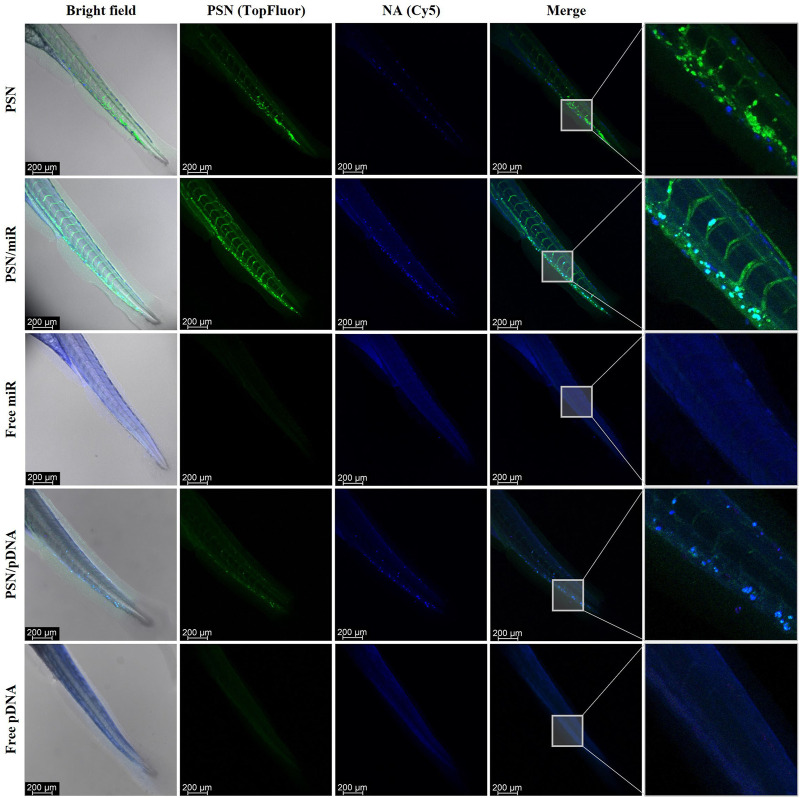
Confocal images of *in vivo* biodistribution of TopFluor-labelled nanoemulsions (green) with and without miR-Cy5 and pDNA (blue), after 48 h incubation in microinjected 48 hpf zebrafish embryos.

For this purpose, 48 hpf embryos were microinjected in the Duct of Cuvier with the PNS, associated and non-associated with NAs (miR and pDNA), as well as free NAs. After 48 h of incubation, embryos were fixed and analyzed by confocal microscopy. The obtained results show the biodistribution of PSN along the zebrafish embryo body through the vasculature and their accumulation in the tail (Figure 4). In the case of PSN associated with NAs (PSN/miR and PSN/pDNA), it is observed that the association between NAs and nanocarriers after the microinjection in circulation is maintained *in vivo*. This maintenance is reflected by the cyan signal observed, which is a result of the co-localization of the green fluorescence of the nanosystems (with SM-TopFluor) and the blue fluorescence from the NAs (labelled with Cy5). Both PSN and PSN associated with NAs display an accumulation pattern that does not appear in the case of the naked miR and pDNA, which are spreading along the zebrafish body.

### 3.5 PSN/miR-pDNA *in vivo* interaction with cancer cells

Another zebrafish embryo property that makes it suitable as an *in vivo* model for gene therapy nanomedicine is the late activation of the immune system, which is not complete until 4–6 wpf (Lam et al., 2004). This allows us to perform xenotransplantation of cancer cells without the necessity of using genetically engineered immunodeficient *in vivo* models. Furthermore, the use of fluorescent-labelled cells, as well as fluorescent PSN and NAs, allows to evaluate how they behave in an *in vivo* tumor-like environment and how they interact with cancer cells.

**FIGURE 5:**
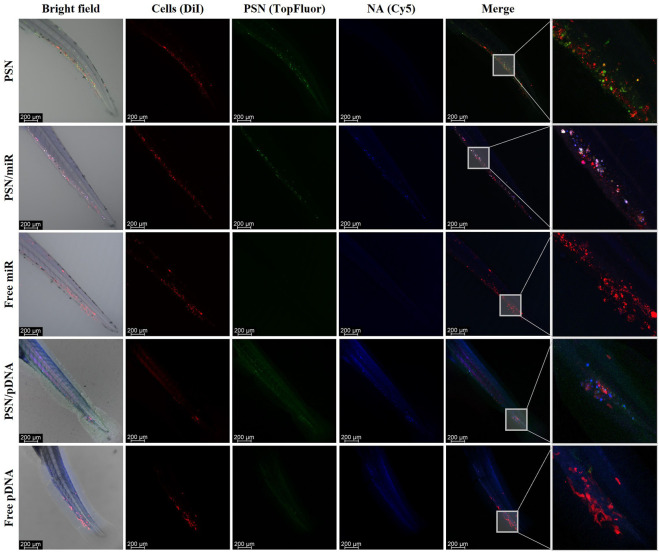
Images of the *in vivo* interaction between nanoemulsions, labelled with TopFluor (green), and associated and non-associated miR and pDNA Cy5-labelled (blue) with DiI-MDA-MB-231 cancer cells (red), by confocal microscopy.

Zebrafish embryos of 48 hpf were microinjected in the Duct of Cuvier with MDA-MB-231 cells, previously labelled with DiI. The result of this injection was a metastasis-like environment with cancer cells spread in the tail of the embryos, a key milieu to evaluate PSN interactions with cancer cells. Twenty-four hours after the xenograft, PSN with SM-TopFluor, associated and non-associated Cy5-NAs, were microinjected in the Duct of Cuvier. Forty-eight hours post-PSN injection, embryos were scanned by confocal microscopy. The results show that the fluorescent signal of the NAs (in blue) co-localize with the PSN (in green), corroborating the results obtained in the biodistribution assay (Figure 5). Further to this, it is observed some co-localization of the fluorescence signal of the PSN (with and without associated NAs) with the fluorescence of cancer cells; whereas free nucleic acids do not show any signal overlapping with the cancer cells. It is also important to highlight that the association between the carrier and the NA is stable 48 h after the microinjection, along the zebrafish circulatory, verifying the stability of the NA-loaded PSN.

## 4 Discussion

Even though zebrafish is widely used as a model to evaluate therapies for cancer treatment (Cascallar et al., 2022; Kwiatkowska et al., 2022), its use to develop and validate innovative gene therapy nanomedicines has not yet been fully investigated. With this aim, this work was carried out to demonstrate the potential of zebrafish as a key model in the study of new gene therapies based on nanotechnology.

Zebrafish is an interesting *in vivo* platform that allows us to perform assays that cannot easily be performed with other *in vivo* model systems, such as rodents. Probably, the most characteristic feature of zebrafish is the transparency present in the embryonic stages (Lieschke & Currie, 2007a; Sieber, Grossen, Bussmann, et al., 2019). As mentioned before, transparency allows the simple visualization of fluorescently labelled molecules, cells, and nanoparticles (Gutiérrez-Lovera et al., 2017; Sieber, Grossen, Bussmann, et al., 2019). This advantage, in synergy with fluorescence/confocal microscopy, permits *in vivo* monitoring of specific structures, such as nanoparticles. As a result, we were able not only to observe how PNS behave *in vivo,* and how they interact with cancer cells, but also to confirm their stability and the maintenance of the association with NAs in an environment similar to that of patients. Our results are in line with several publications that use zebrafish as a model to evaluate nanomedicines (not for gene therapy purposes) and demonstrate how zebrafish can be used to evaluate novel cationic lipidic nanoemulsions *in vivo* with associated NAs (Sieber, Grossen, Bussmann, et al., 2019; Saez Talens et al., 2020; Rességuier et al., 2021; Cascallar et al., 2022).

Although zebrafish transparency plays a key role in carrying out these types of assays, this is not the only interesting advantage of this model system. Both embryos and adults have a small size and can be easily stored and maintained. This characteristic allows cost-effective, large scale assays with a large number of specimens, with enough replicates to validate the experiments (Lieschke & Currie, 2007b; Delvecchio et al., 2011; Veinotte et al., 2014). These types of assays are inconceivable in mice, considering the high maintenance cost and the small number of progeny (Veinotte et al., 2014). In addition, zebrafish genome has a great homology with the human genome, and the body with several vertebrate structures (Lieschke & Currie, 2007b; Delvecchio et al., 2011).

Certainly, zebrafish embryo has several advantages that make it suitable as a model platform to evaluate cancer nanotechnology-based therapies, resulting in a plethora of diverse experiments that can be done to optimize and select the best treatments. However, it also has limitations in terms of similarity with humans. For instance, the lack of the physiological complexity of a non-mammalian organism implies the need to combine the zebrafish with other models, such as mice and rats, in certain types of experiments. However, in the context of animal welfare, combining the use of zebrafish embryo with more complex models may help to implement the 3R’s rule: replace, reduce, and refine (MacArthur Clark, 2018; Lewis, 2019). Because the zebrafish is an intermediate model between cell cultures and rodents, all experiments that can be performed in zebrafish models would inversely affect the number of mice that will be needed in subsequent experiments.

Our group has previously developed a new type of cationic nanosystems composed by Vitamin E, Sphingomyelin and a Putrescine derivative, PSN (Lores et al., 2022). This formulation is an optimization of previous sphingomyelin nanosystems (Bouzo et al., 2020; Nagachinta et al., 2020; Bidan et al., 2022), for cancer gene therapy applications, taking advantage of the intrinsic properties of putrescine. Among others, putrescine provides a cationic charge that can establish electrostatic interactions with negative-charged molecules such as NAs (Rowe et al., 2009; Chakraborty and Jiang, 2013; Agostinelli et al., 2015). In addition, cancer cells show a higher affinity for putrescine compared to normal cells, in order to maintain their metabolic activities, in which natural polyamines are involved (Tracy et al., 2016; Casero et al., 2018). Our previous results show the potential of PSN to efficiently carry anti-cancer therapeutic pDNA, achieving a therapeutic effect in cell culture. Most importantly, the results show a tumor reduction in murine models of cancer, which correlates with the reduction previously observed in xenografted zebrafish embryos (Lores et al., 2022).

Furthermore, the experiments performed in the present work allowed us to validate the adaptability of our PSN cationic nanoemulsions (Lores et al., 2022). PSN demonstrated to be an innovative carrier for gene therapy showing a high versatility due to their ability to carry different types of NAs, not only with a huge difference in length but also with different nature, desoxyribonucleic (pDNA) and ribonucleic acids (miR). Moreover, results obtained in 0 hpf embryos confirmed that miR145 is able to develop its regulatory role in zebrafish genes when associated with nanoemulsions; and therefore, the PSN are capable of releasing their cargo inside the cell. This fact highlights the potential of zebrafish to study the transfection efficiency of gene delivery nanosystems. It is important to emphasize that this type of experiment, with 0 hpf embryos, cannot be performed in the common models used in experimentation, such as mice and rats. These models, with higher complexity, have internal fertilization, thus this assay becomes complicated by the need to perform *in vitro* fecundation.

The accumulation of PSN observed in tumor cells could be related to the incorporation of putrescine and the fact that polyamine uptake is increased in cancer cells through Polyamine Transport Systems (Novita Sari et al., 2021). Importantly, we observed that PSN were stable in an *in vivo* environment, maintaining an efficient association of the NAs, which were then successfully released inside the cells. This proved the ability of putrescine to protect the NAs against *in vivo* barriers due to its capacity to condense nucleic acids achieving an improvement in the transport inside the cells (Thomas et al., 2016). In this sense, zebrafish allow us to observe/visualize the interaction of PSN with cancer cells in a more complex system than the one represented by a cell culture since different cell types from the tumor environment are present in the fish. Interaction studies verified that PSN/miR-pDNA are able to travel along the embryos and reach the cancer cells. These results corroborate the ones observed in uptakes performed in cancer cell lines, demonstrating that the PSN interaction with cancer cells happens both *in vitro* and *in vivo* (Supplementary Figures S2,S3). In other words, these results confirm that we have developed an efficient and stable nanocarrier able to transport its cargo to the cancer cells. Furthermore, the working times between cell cultures and zebrafish embryos are quite similar, obtaining more complex and reliable results.

In conclusion, our results demonstrate the huge potential that zebrafish embryos have as an *in vivo* platform to evaluate nanomedicines for gene therapy in a fast, cost-effective and reliable way in contrast with other animal models. In the same vein, the experiments presented here validated the capacity of PSN to successfully associate and transport different types of NAs into a living organism.

## Data Availability

The original contributions presented in the study are included in the article/Supplementary Material, further inquiries can be directed to the corresponding author.
