# Supplementary figures and images for: Zebrafish as a platform to evaluate the potential of lipidic nanoemulsions for gene therapy in cancer
Source: Front Pharmacol. 2022 Oct 31;13:1007018. doi: 10.3389/fphar.2022.1007018 (PMC9659613; doi:10.3389/fphar.2022.1007018)

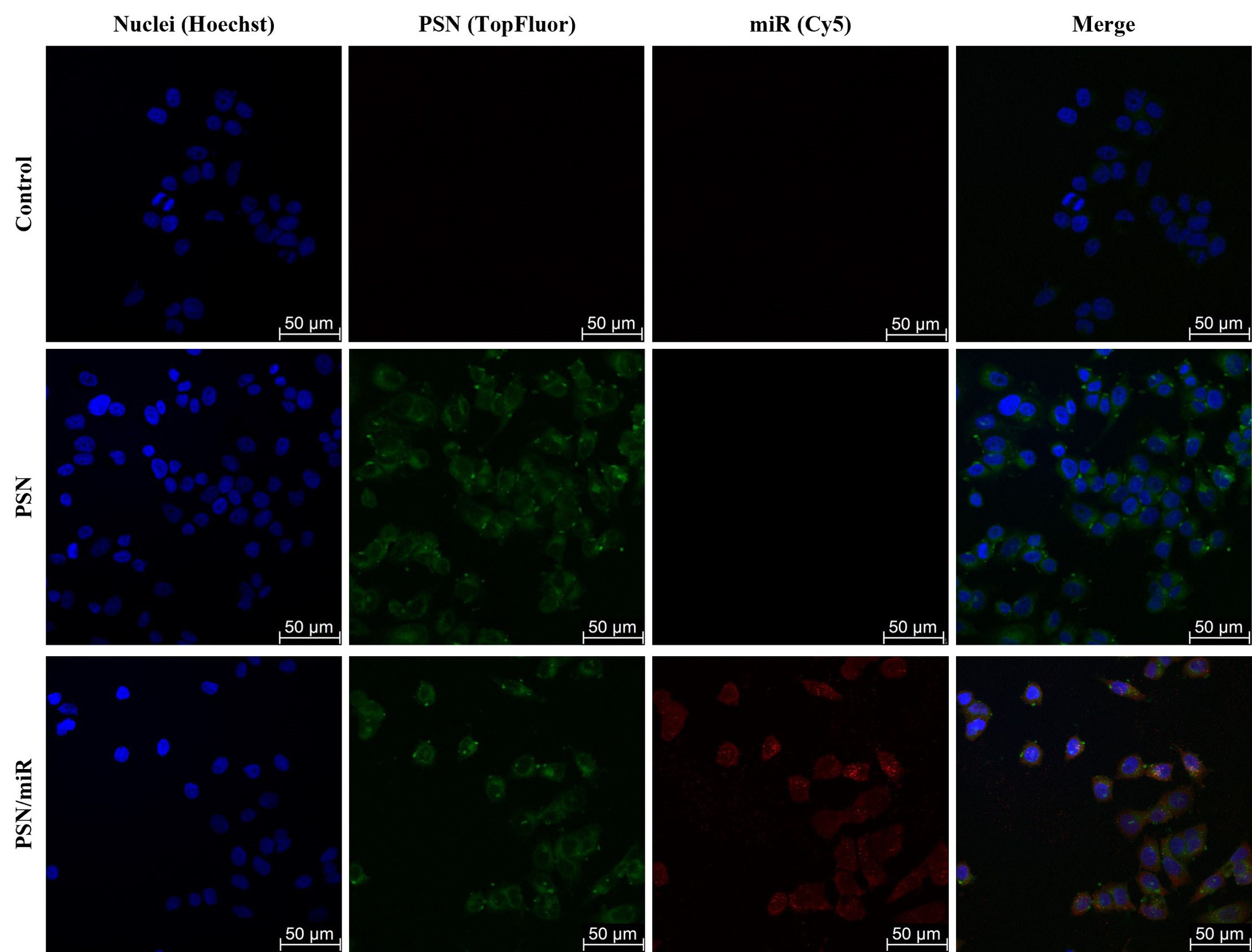

Supplement: Supplementary file 1 [file Image3.JPEG]

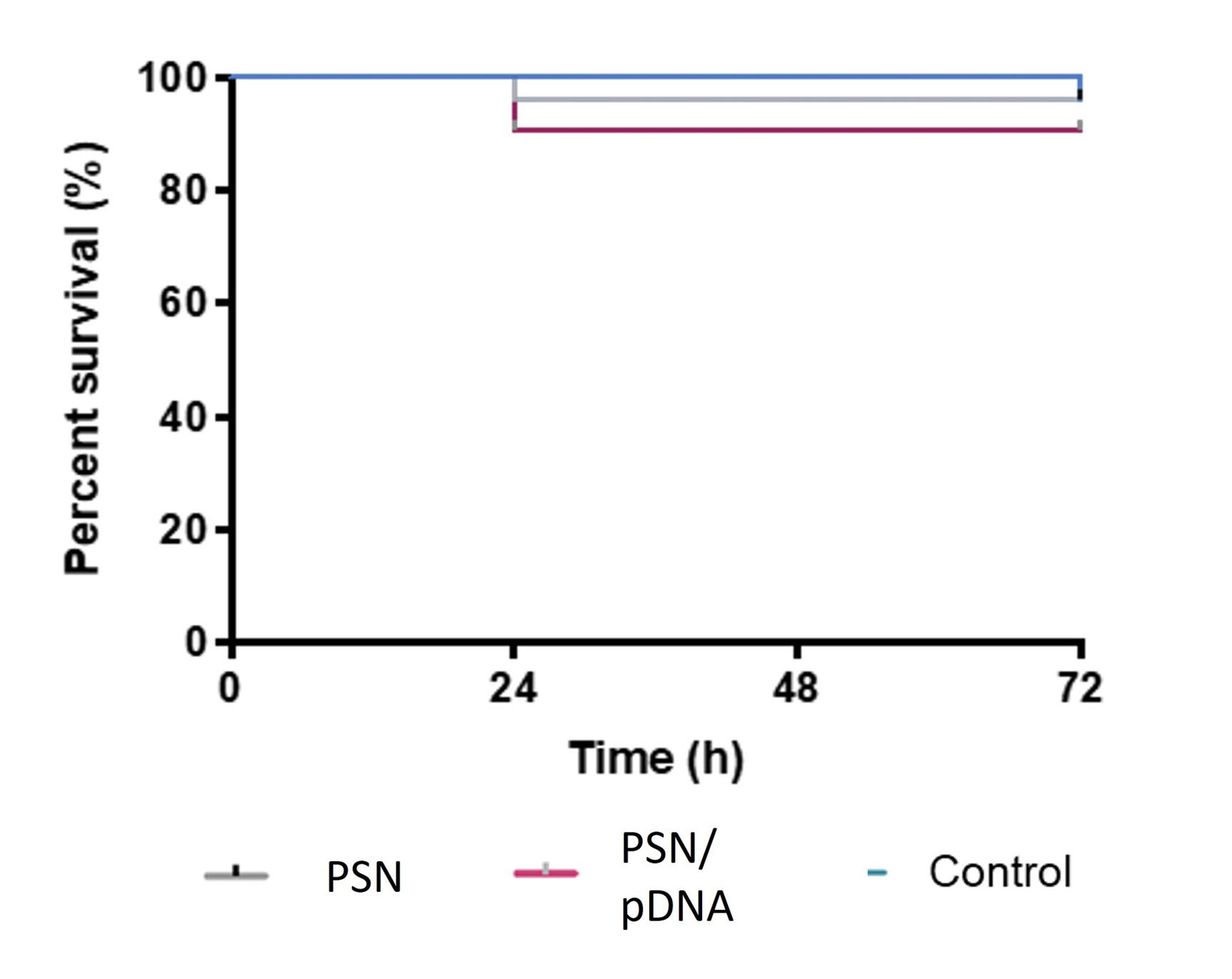

Supplement: Supplementary file 2 [file Image1.JPEG]

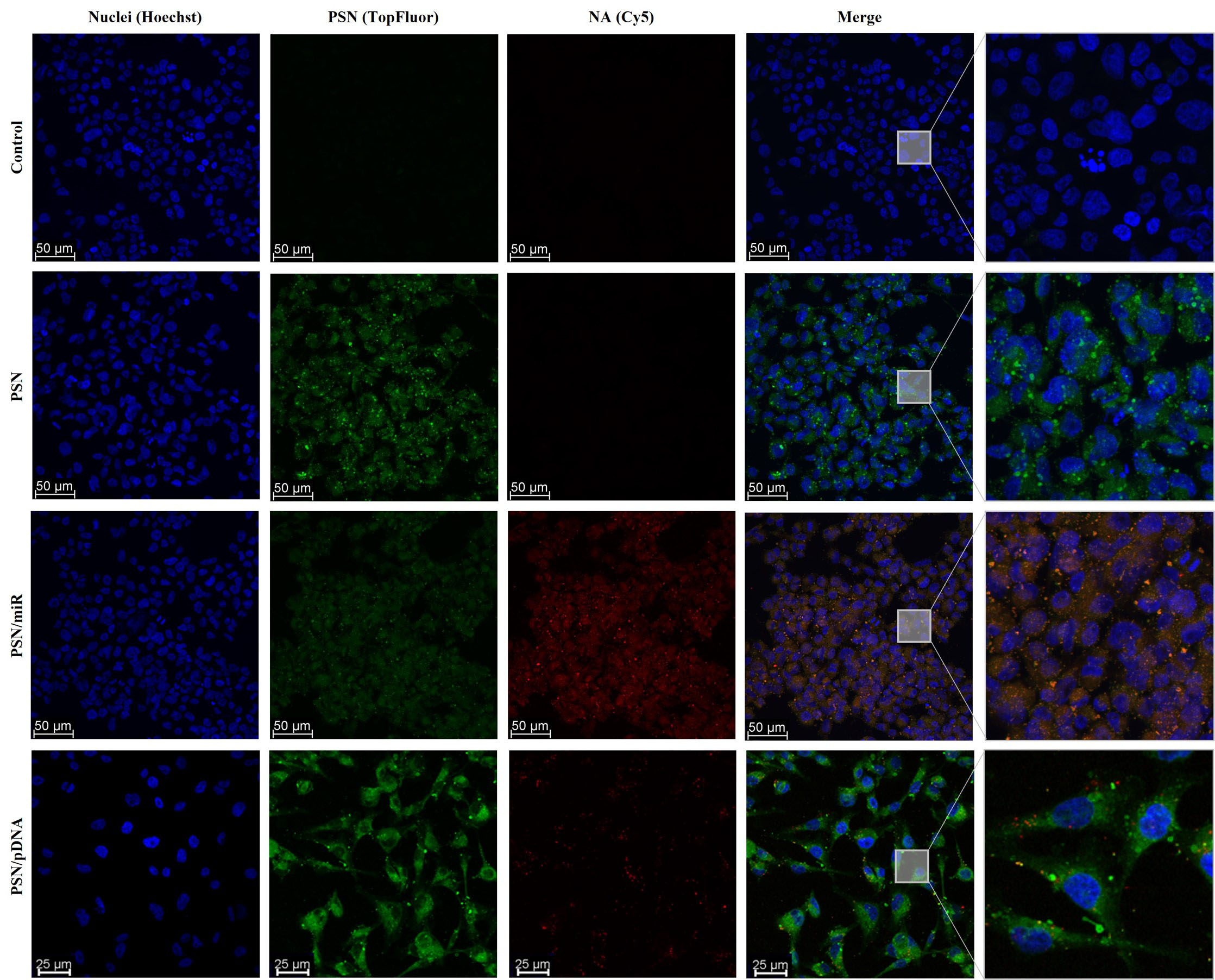

Supplement: Supplementary file 3 [file Image2.JPEG]
