# Supplementary material for: Zebrafish as a platform to evaluate the potential of lipidic nanoemulsions for gene therapy in cancer
Source: Front Pharmacol. 2022 Oct 31;13:1007018. doi: 10.3389/fphar.2022.1007018 (PMC9659613; doi:10.3389/fphar.2022.1007018)
Supplement: Supplementary file 4 [file DataSheet1.docx]

Supplementary Material

**
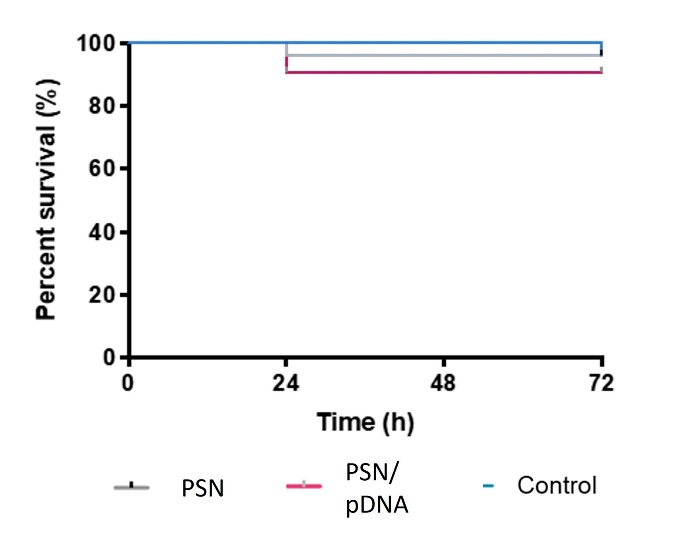
**

**Figure S1.** Toxicity assay of PSN and PSN/pDNA in zebrafish embryos after 24, 48 and 72 hours of incubation.


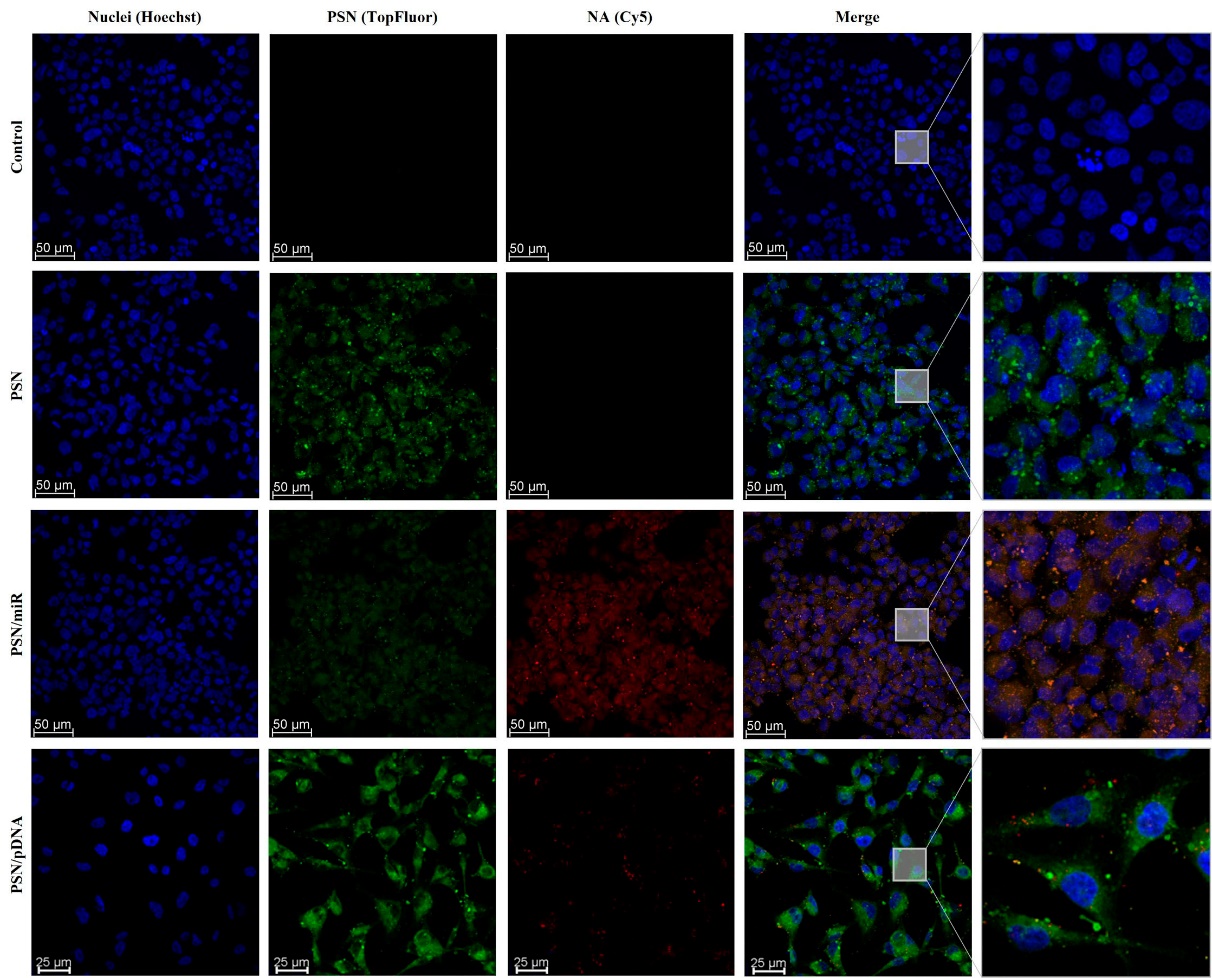


**Figure S2.** Confocal images of triple negative breast cancer cells (MDA-MB-231 cell line) after a 4h incubation with TopFluor-labelled nanosystems (green) associated and non-associated with miR/pDNA-Cy5 (red). Cell nuclei were stained with Hoescht (blue).


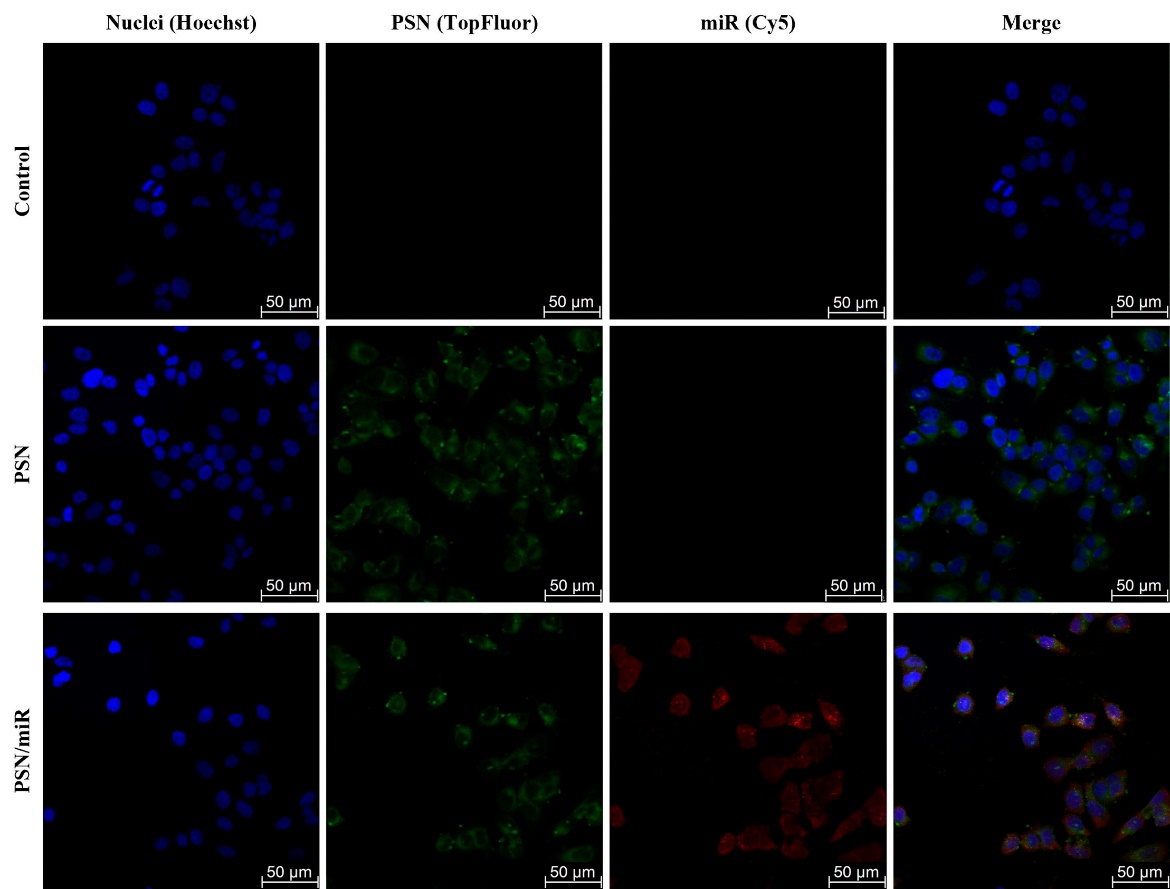
**Figure S3.** Confocal images of MCF7 breast cancer cells resulting after a 4h incubation with nanocarriers labelled with TopFluor (green) associated and non-associated with miR-Cy5 (red). Cell nuclei were stained with Hoescht (blue).
